# Supplementary material for: Modeling glioblastoma heterogeneity as a dynamic network of cell states
Source: Mol Syst Biol. 2021 Sep 16;17(9):e10105. doi: 10.15252/msb.202010105 (PMC8444284; doi:10.15252/msb.202010105)
Supplement: Supplementary file 5 — Source Data for Figure 3 [file MSB-17-e10105-s001.zip › Figure3A_sourcedata/GSEA_3065/nosigdb_state1.GseaPreranked.1622718409535/RICHARDS_VERHAAK_CANCERCELL_2010_MESENCHYMAL.html]

Details for gene set RICHARDS\_VERHAAK\_CANCERCELL\_2010\_MESENCHYMAL[GSEA]

|  || Dataset | state1 |
| Phenotype | NoPhenotypeAvailable |
| Upregulated in class | na\_pos |
| GeneSet | RICHARDS\_VERHAAK\_CANCERCELL\_2010\_MESENCHYMAL |
| Enrichment Score (ES) | 0.5330756 |
| Normalized Enrichment Score (NES) | 1.9365839 |
| Nominal p-value | 0.0 |
| FDR q-value | 3.9418845E-4 |
| FWER p-Value | 0.006 |
Table: GSEA Results Summary

  

Fig 1: Enrichment plot: RICHARDS\_VERHAAK\_CANCERCELL\_2010\_MESENCHYMAL      
 Profile of the Running ES Score & Positions of GeneSet Members on the Rank Ordered List

  

| PROBE | GENE SYMBOL | GENE\_TITLE | RANK IN GENE LIST | RANK METRIC SCORE | RUNNING ES | CORE ENRICHMENT || 1 | LGALS1 |  |  | 4 | 0.828 | 0.0523 | Yes |
| 2 | S100A13 |  |  | 20 | 0.627 | 0.0907 | Yes |
| 3 | ANXA1 |  |  | 30 | 0.564 | 0.1257 | Yes |
| 4 | CAST |  |  | 35 | 0.534 | 0.1593 | Yes |
| 5 | CTSC |  |  | 43 | 0.498 | 0.1903 | Yes |
| 6 | ANXA2 |  |  | 48 | 0.482 | 0.2206 | Yes |
| 7 | S100A11 |  |  | 51 | 0.475 | 0.2507 | Yes |
| 8 | DCBLD2 |  |  | 62 | 0.444 | 0.2779 | Yes |
| 9 | CLIC1 |  |  | 76 | 0.406 | 0.3025 | Yes |
| 10 | ARPC1B |  |  | 108 | 0.358 | 0.3221 | Yes |
| 11 | ARSJ |  |  | 114 | 0.348 | 0.3438 | Yes |
| 12 | MYOF |  |  | 115 | 0.348 | 0.3660 | Yes |
| 13 | LGALS3 |  |  | 117 | 0.348 | 0.3881 | Yes |
| 14 | ARHGAP29 |  |  | 120 | 0.346 | 0.4099 | Yes |
| 15 | MYH9 |  |  | 140 | 0.328 | 0.4288 | Yes |
| 16 | FHL2 |  |  | 141 | 0.328 | 0.4497 | Yes |
| 17 | PLS3 |  |  | 167 | 0.312 | 0.4670 | Yes |
| 18 | RRAS |  |  | 207 | 0.292 | 0.4816 | Yes |
| 19 | CNN2 |  |  | 208 | 0.291 | 0.5001 | Yes |
| 20 | VAMP5 |  |  | 246 | 0.278 | 0.5140 | Yes |
| 21 | RAB32 |  |  | 351 | 0.239 | 0.5186 | Yes |
| 22 | LRRFIP1 |  |  | 399 | 0.226 | 0.5282 | Yes |
| 23 | COL1A2 |  |  | 517 | 0.201 | 0.5290 | Yes |
| 24 | TGFBR2 |  |  | 650 | 0.179 | 0.5269 | Yes |
| 25 | BLVRB |  |  | 945 | 0.140 | 0.5057 | Yes |
| 26 | ACSL1 |  |  | 962 | 0.138 | 0.5129 | Yes |
| 27 | WWTR1 |  |  | 1009 | 0.133 | 0.5167 | Yes |
| 28 | TNFRSF1B |  |  | 1031 | 0.130 | 0.5228 | Yes |
| 29 | THBS1 |  |  | 1118 | 0.121 | 0.5217 | Yes |
| 30 | RAB27A |  |  | 1182 | 0.114 | 0.5225 | Yes |
| 31 | SERPINE1 |  |  | 1250 | 0.108 | 0.5225 | Yes |
| 32 | ANXA4 |  |  | 1348 | 0.099 | 0.5189 | Yes |
| 33 | RBMS1 |  |  | 1377 | 0.097 | 0.5222 | Yes |
| 34 | SHC1 |  |  | 1393 | 0.096 | 0.5268 | Yes |
| 35 | PLAUR |  |  | 1463 | 0.091 | 0.5255 | Yes |
| 36 | CEBPB |  |  | 1680 | 0.076 | 0.5083 | Yes |
| 37 | NRP1 |  |  | 1691 | 0.076 | 0.5121 | Yes |
| 38 | GCNT1 |  |  | 1696 | 0.076 | 0.5165 | Yes |
| 39 | SP100 |  |  | 1721 | 0.074 | 0.5187 | Yes |
| 40 | RHOG |  |  | 1768 | 0.071 | 0.5185 | Yes |
| 41 | UAP1 |  |  | 1780 | 0.071 | 0.5219 | Yes |
| 42 | PLAU |  |  | 1801 | 0.069 | 0.5243 | Yes |
| 43 | LOX |  |  | 1825 | 0.068 | 0.5263 | Yes |
| 44 | CD2AP |  |  | 1827 | 0.068 | 0.5305 | Yes |
| 45 | UCP2 |  |  | 1845 | 0.067 | 0.5331 | Yes |
| 46 | P4HA2 |  |  | 1910 | 0.065 | 0.5306 | No |
| 47 | COPZ2 |  |  | 1990 | 0.060 | 0.5264 | No |
| 48 | CASP4 |  |  | 2054 | 0.057 | 0.5235 | No |
| 49 | ICAM3 |  |  | 2068 | 0.056 | 0.5258 | No |
| 50 | S100A4 |  |  | 2114 | 0.054 | 0.5247 | No |
| 51 | PROCR |  |  | 2129 | 0.054 | 0.5266 | No |
| 52 | SLC10A3 |  |  | 2184 | 0.052 | 0.5244 | No |
| 53 | LAMB1 |  |  | 2262 | 0.049 | 0.5196 | No |
| 54 | MAN2A1 |  |  | 2270 | 0.048 | 0.5220 | No |
| 55 | ASL |  |  | 2286 | 0.048 | 0.5235 | No |
| 56 | ITGA5 |  |  | 2302 | 0.047 | 0.5249 | No |
| 57 | RELB |  |  | 2338 | 0.046 | 0.5243 | No |
| 58 | ZNF217 |  |  | 2460 | 0.041 | 0.5145 | No |
| 59 | TIMP1 |  |  | 2497 | 0.040 | 0.5133 | No |
| 60 | SRPX2 |  |  | 2578 | 0.037 | 0.5075 | No |
| 61 | PHF11 |  |  | 2588 | 0.037 | 0.5089 | No |
| 62 | IQGAP1 |  |  | 2622 | 0.035 | 0.5078 | No |
| 63 | TRIM38 |  |  | 2645 | 0.035 | 0.5077 | No |
| 64 | TRIM22 |  |  | 2675 | 0.034 | 0.5069 | No |
| 65 | CASP8 |  |  | 2688 | 0.033 | 0.5078 | No |
| 66 | SH2B3 |  |  | 2725 | 0.032 | 0.5062 | No |
| 67 | ITGA4 |  |  | 2746 | 0.032 | 0.5061 | No |
| 68 | TRADD |  |  | 2869 | 0.028 | 0.4954 | No |
| 69 | RUNX2 |  |  | 3110 | 0.022 | 0.4723 | No |
| 70 | EHD2 |  |  | 3127 | 0.022 | 0.4720 | No |
| 71 | MVP |  |  | 3193 | 0.020 | 0.4667 | No |
| 72 | FHOD1 |  |  | 3408 | 0.016 | 0.4458 | No |
| 73 | YAP1 |  |  | 3463 | 0.015 | 0.4412 | No |
| 74 | TGFBI |  |  | 3688 | 0.010 | 0.4189 | No |
| 75 | FURIN |  |  | 3725 | 0.009 | 0.4158 | No |
| 76 | TNFAIP8 |  |  | 3901 | 0.006 | 0.3983 | No |
| 77 | SYNGR2 |  |  | 4130 | 0.002 | 0.3750 | No |
| 78 | SEC24D |  |  | 4162 | 0.001 | 0.3719 | No |
| 79 | MGAT1 |  |  | 4228 | 0.000 | 0.3653 | No |
| 80 | LY96 |  |  | 4335 | -0.002 | 0.3545 | No |
| 81 | ALDH3B1 |  |  | 4547 | -0.005 | 0.3333 | No |
| 82 | DRAM1 |  |  | 4678 | -0.007 | 0.3204 | No |
| 83 | TMBIM1 |  |  | 4715 | -0.008 | 0.3172 | No |
| 84 | PIGP |  |  | 4785 | -0.009 | 0.3107 | No |
| 85 | CTSB |  |  | 4873 | -0.011 | 0.3025 | No |
| 86 | SWAP70 |  |  | 4882 | -0.011 | 0.3023 | No |
| 87 | PLK3 |  |  | 5051 | -0.013 | 0.2860 | No |
| 88 | LHFPL2 |  |  | 5149 | -0.015 | 0.2770 | No |
| 89 | DSE |  |  | 5179 | -0.015 | 0.2750 | No |
| 90 | MGST2 |  |  | 5294 | -0.017 | 0.2644 | No |
| 91 | RABGAP1L |  |  | 5486 | -0.020 | 0.2461 | No |
| 92 | MFSD1 |  |  | 6232 | -0.034 | 0.1719 | No |
| 93 | WIPF1 |  |  | 7001 | -0.050 | 0.0964 | No |
| 94 | CYBRD1 |  |  | 7284 | -0.058 | 0.0712 | No |
| 95 | PLA2G15 |  |  | 7324 | -0.059 | 0.0710 | No |
| 96 | FNDC3B |  |  | 7392 | -0.060 | 0.0680 | No |
| 97 | EMP3 |  |  | 7404 | -0.061 | 0.0707 | No |
| 98 | CPQ |  |  | 7511 | -0.064 | 0.0639 | No |
| 99 | SCPEP1 |  |  | 7585 | -0.066 | 0.0606 | No |
| 100 | COL5A1 |  |  | 7642 | -0.067 | 0.0592 | No |
| 101 | EFEMP2 |  |  | 7651 | -0.068 | 0.0627 | No |
| 102 | PYGL |  |  | 8196 | -0.089 | 0.0126 | No |
| 103 | COLGALT1 |  |  | 8276 | -0.093 | 0.0105 | No |
| 104 | TNFRSF1A |  |  | 8416 | -0.100 | 0.0026 | No |
| 105 | FCGR2A |  |  | 8506 | -0.105 | 0.0002 | No |
| 106 | SYPL1 |  |  | 8546 | -0.108 | 0.0031 | No |
| 107 | SFT2D2 |  |  | 8664 | -0.115 | -0.0016 | No |
| 108 | ADAM12 |  |  | 8684 | -0.116 | 0.0039 | No |
| 109 | HEXB |  |  | 8966 | -0.140 | -0.0160 | No |
| 110 | NPC2 |  |  | 9235 | -0.170 | -0.0326 | No |
| 111 | PDPN |  |  | 9284 | -0.177 | -0.0262 | No |
| 112 | TGOLN2 |  |  | 9316 | -0.184 | -0.0177 | No |
| 113 | HEXA |  |  | 9438 | -0.208 | -0.0168 | No |
| 114 | GRN |  |  | 9593 | -0.257 | -0.0162 | No |
| 115 | MRC2 |  |  | 9620 | -0.266 | -0.0019 | No |
| 116 | SAT1 |  |  | 9807 | -0.445 | 0.0074 | No |
Table: GSEA details [plain text format]

  

Fig 2: RICHARDS\_VERHAAK\_CANCERCELL\_2010\_MESENCHYMAL: Random ES distribution      
 Gene set null distribution of ES for **RICHARDS\_VERHAAK\_CANCERCELL\_2010\_MESENCHYMAL**
